# Stiffness Modulation in Flexible Rotational Triboelectric Nanogenerators for Dual Enhancement of Power and Reliability

**DOI:** 10.3390/nano14040380

**Published:** 2024-02-18

**Authors:** Feng Li, Ao Yin, Yaao Zhou, Tao Liu, Qingqing Liu, Weijie Ruan, Ling Bu

**Affiliations:** 1 School of Information Engineering, China University of Geosciences, Beijing 100083, China; 2104200005@cugb.edu.cn (F.L.); 2104230066@email.cugb.edu.cn (A.Y.); 2004220003@email.cugb.edu.cn (T.L.); 2104230012@email.cugb.edu.cn (Q.L.); 1004222124@email.cugb.edu.cn (W.R.); 2 Power Transmission and Substation Department, China Electric Power Research Institute, Beijing 100055, China; zhouyaao@epri.sgcc.com.cn

**Keywords:** stiffness modulation, flexible, rotational triboelectric nanogenerators, reliability

## Abstract

Rotational nanogenerators with flexible triboelectric layers have wide applications and high reliability. However, flexible materials cause a severe reduction in contact force and thus triboelectric output power. Unlike previous works devising complex auxiliary structures to solve this issue, this paper focuses on improving the contact material mechanics and proposes a stiffness modulation method. By introducing fine patterns to the contacting rotor–stator pairs, the effective elastic modulus was regulated from approximately 10^3^ to 10^5^ MPa, and the output voltage was modulated from approximately 24.39% to 375.87% compared to the non-patterned rotor–stator pairs, corresponding to a maximal a 14 times increase in output power. A maximal power density of 18.75 W/m^2^ was achieved on 10 MΩ resistance at 9.6 Hz, which is even beyond the power density of most rigid triboelectric interfaces. Moreover, high reliability could be maintained when the volume ratio of the horizontal patterns exceeded a threshold value of 33.5% as the stator and 63.6% as the rotor for a 0.5 mm linewidth. These results prove the efficacy of the stiffness modulation method for jointly achieving high output power and high reliability in flexible rotational triboelectric nanogenerators.

## 1. Introduction

Rotational triboelectric nanogenerators (R-TENGs) have promising potential in fields like wearable electronics [1,2,3], meteorological monitoring [4,5,6], blue energy [7,8,9], etc. Early works adopted disc-shaped and multilayered structures, where the rigid triboelectric layers are pressed face to face, and charges are generated through the lateral sliding mode. Zhu et al. proposed a radial-arrayed R-TENG featuring micro-sized spokes, achieving a peak area power density of 19 mW/cm^2^ at a rotation speed of 3000 rpm [10]. Gao et al. designed an R-TENG utilizing free-standing triboelectrification between the stator and rotor with arrays of radial sectors, generating a maximum power density of 2.28 W/m^2^ at 600 rpm [11]. Chen et al. fabricated an R-TENG based on a double friction layer rotating disc structure, producing a peak power density of 1.36 W/m^2^ at a rotation speed of 600 rpm [12]. Generally, these disc-type R-TENGs generate high output power due to their large contact area and effective triboelectrification but require a strong driving force to overcome the sliding friction, which typically results in severe abrasion of the triboelectric layers [13,14,15,16].

Recently, flexible triboelectric nanogenerators have received considerable attention due to their versatile applications in electronic skin [17,18,19], tactile human–machine interfaces [20], humidity detection [21], electrochemical systems [22], etc. The superb deformability of flexible materials significantly enhances the reliability of the triboelectric layers, and makes them highly compatible with most complex surfaces. Flexible rotation triboelectric nanogenerators (FR-TENGs) can be more agilely incorporated into applications like body moving [23], button pressing [24], machine lathing [25], geological drilling [26,27,28], etc. However, the contact force between flexible triboelectric layers is much smaller than that between their rigid counterparts, which is a key limiting factor for the relatively low output power of FR-TENGs.

To improve the output power of FR-TENGs, diverse design technologies have been brought forward. One method involves coupling multiple transduction mechanisms to form hybrid TENGs. Rahman et al. reported a hybrid nanogenerator combining triboelectric, piezoelectric, and electromagnetic mechanisms. The triboelectric part utilized flexible blades and generated a maximum power of 1.67 mW at the wind speed of 6 m/s, while the other two parts generated a maximal power of 269.98 mW altogether [29]. Zhao et al. incorporated a bimorph-based piezoelectric nanogenerator into the FR-TENG to construct a hybrid piezoelectric/triboelectric nanogenerator for efficient mechanical rotation energy harvesting. At a wind speed of 14 m/s, the average power of the FR-TENG was 1.95 mW, while that of the piezoelectric part was 8.67 mW [30]. Although hybrid nanogenerators generate higher total power, the partial output power generated by the FR-TENG is still relatively low. Moreover, coupling multiple mechanisms increases both the design complexity and device volume of the nanogenerators.

The other method involves designing auxiliary contact structures of FR-TENGs for higher output power. Liu et al. designed a novel TENG with vortex-like flexible self-recovery blades on the inner stator and a rotating outer rotor. By optimizing the number, thickness, and area of the flexible blades, their proposed TENG exhibited an instantaneous short-circuit current of 350 μA and open-circuit voltage of 650 V [31]. Zhang et al. developed a non-contact cylindrical TENG. Operation of the TENG was based on the non-contact free rotation between a curved Cu foil and a flexible nanostructured fluorinated ethylene propylene polymer film. When driven by water flow, the output voltage and current of the TENG reached 1670 V and 13.4 μA, respectively [32]. Men et al. designed a soft-contact flower-bud-array cotton-based TENG. The adopted double stator–rotor structure was cellulose-rich, cotton-assisted, and soft-contact, which increased the contact area, and it reached an open-circuit voltage of 2500 V, a short circuit current of 85 μA, and a maximum output power of 80 mW [33]. Cao et al. fabricated a rotary electrode-less TENG using the contact and sliding mode with segmented structure, consisting of a polypropylene (PP) disc and polymethyl methacrylate (PMMA) sectors, which rotated clockwise around the central axis to change the spatial electric field periodically. At a rotating speed of 1000 rpm, the output current density reached about 7 µA/cm^2^ [34]. Lin et al. introduced a non-contact, free-rotating disc TENG consisting of an independent rotating part for the fluorinated ethylene propylene (FEP) layer and a fixed part for the aluminum foil electrodes with complementary shapes. The device contacted the FEP with two separate layers of aluminum foil to generate an output power density of 1.22 W/m^2^ [35]. Though these designs improve the output power, they require complex or even special structures that prove difficult when catering for versatile applications.

In this paper, an alternative method of stiffness modulation is proposed to increase the contact force while not sacrificing the high reliability of FR-TENGs. Inspired by the microstructures in fiber/matrix composite materials, we propose the elastic modulus modulation of the flexible electrodes by controlling the pattern alignment and volume fraction, thus altering the stiffness of the flexible electrodes. Unlike those described in previous reports, this method concentrates on improving the material mechanical properties of the contact structure and therefore avoids complex auxiliary structure design. An FR-TENG with modulated stator/rotor stiffness was designed, fabricated, and tested. By comparing the output power with different stator/rotor stiffnesses, the optimal stiffness combination was verified, proving that stiffness modulation is an effective method for increasing the contact force and output power while maintaining the reliability of the FR-TENG.

## 2. Methods

### 2.1. Fabrication of Rotors and Stators

Polytetrafluoroethylene (PTFE) skived film tape (3M^TM^, 5480) and 304 stainless steel (SS) were selected as the friction materials for FR-TENG. The PTFE film was 90 μm thick. The elastic modulus of the PTFE tape was approximately 550 MPa, negligible compared with that of SS, 195 GPa. The SS material that was 100 μm thick was fabricated by laser ablation to form the horizontal and vertical patterns with linewidths of 0.2 mm and 0.5 mm, respectively. For each linewidth, six volume ratios ranging from approximately 18.4% to 88.9% were fabricated, so altogether 24 SS samples were prepared in this experiment. The volume ratio *R* is defined as:

(1) $$R=\frac{{Volume}_{fiber}}{{Volume}_{void}}$$

where *Volume_fiber_* represents the volume of fibers, and *Volume_void_* represents the volume with no fibers. The dimensions of the PTFE films and SS samples were 27 × 10 mm^2^ and 30 × 10 mm^2^, respectively. The stators were formed by PTFE film stuck onto the SS sample surface, simplified as SS/PTFE stators. The rotor only consisted of the SS sample, simplified as SS rotors.

### 2.2. Assembly of FR-TENG Devices

A circular polymethyl methacrylate (PMMA) fixture with diameter of 13.9 cm was fabricated by computer numerical control (CNC) machining. The stator was one-end-fixed at the rim of the circular fixture, and the SS part was electrically connected to record the generated electrical signal. The rotor was one-end-fixed to the DC motor shaft through a connector and was electrically grounded. The DC motor was fixed inside the circular fixture and was driven by a DC voltage source. The proposed FR-TENG enables a maximum of 8 stators and 3 rotors to be synchronously connected. Both the circular rim and the motor were secured on the testing platform.

### 2.3. Characterization and Measurements

The rotation speed of the motor was controlled by a DC voltage source (Zhaoxin MN-3205D, Shenzhen, China) and was measured using a photoelectric tachometer (VICTOR DM6234P+, Shenzhen, China). A locked-in amplifier (Stanford 865 A, Sunnyvale, CA, USA) and an electronic balance (WANTE 3003, Zhejiang, China) were adopted to measure the first mode resonant frequency and the mass of specific SS samples. The generated electrical signals on the flexible stator were measured using the oscilloscope (Keysight InfiniiVision DSOX2024A, Santa Rosa, CA, USA) with 1 MΩ input resistance.

## 3. Results and Discussion

### 3.1. Design and Implementation

**Figure 1:**
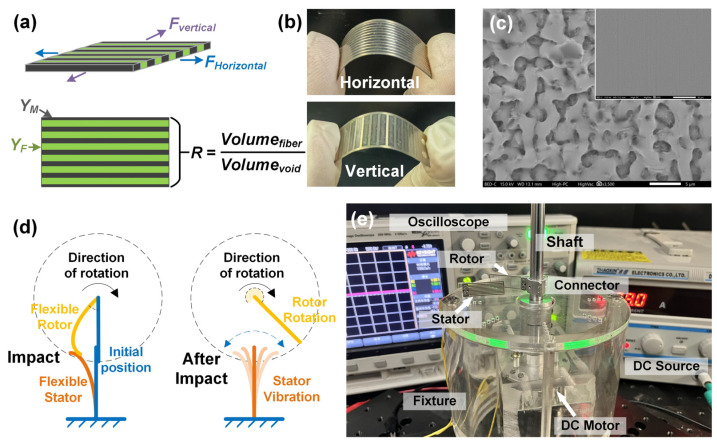
Device design, fabrication, and experimental setup: (**a**) stiffness modulation through matrix–fiber composite materials. (**b**) Photo of fabricated flexible stator/rotor with horizontal and vertical patterns. The linewidth in the photos is 500 μm. (**c**) Surface morphology of the O_2_-plasma-treated PTFE film with a bar of 5 μm. The inset shows the generally flat PTFE film with a bar of 50 μm. (**d**) Schematic plot of rotor and stator in impact and after impact. (**e**) Photo of the assembled FR-TENG with one stator and one rotor mounted on the testing bench.

The device design, fabrication, and assembly are shown in Figure 1. The design concept of stiffness modulation is based on the elastic modulus modulation theory of matrix/fiber composite materials. As shown in Figure 1a, we denote the elastic modulus of the fibers as *Y_F_* and that of the matrix as *Y_M_.* The fiber is of a higher stiffness than the matrix. Adjusting the fiber volume ratio *R*, the elastic modulus of the composite materials accordingly changes.

Moreover, applying a different direction of tension force also results in a different effective elastic modulus. Supposing *E_H_* is the effective elastic modulus when tension force is parallel to fiber length, and *E_V_* is the effective elastic modulus when tension force is perpendicular to fiber length, *E_H_* and *E_V_* can be described as:

(2) $$E_{H}=Y_{F}\frac{R}{1+R}+Y_{M}\frac{R}{1+R}$$

(3) $$E_{V}=\frac{Y_{F}Y_{M}\left(1+R\right)}{Y_{F}+Y_{M}R}$$

If the composite materials are adopted as a cantilever, a change in the elastic modulus results in a change in the cantilever first-mode resonant frequency *f*, as indicated in Equation (4):

(4) $$f=0.56{\left(\frac{EI}{\rho Al^{4}}\right)}^{\frac{1}{2}}$$

where *E* is the effective elastic modulus, *I* is the section moment of inertia, *A* is the cross-sectional area of the beam, ρ and l, respectively, represent the density and length of the beam. 

The modulated stiffness for each stator is further derived as:

(5) $$k=4\pi^{2}mf^{2}$$

where *m* is the mass of the cantilever.

To implement the above design concept, patterned stainless steel (SS) samples were adopted to serve as the fibers with high stiffness. A layer of PTFE film was pasted onto the patterned SS samples to serve as the matrix with low stiffness. The SS/PTFE comprised the fiber/matrix composite system with good flexibility, as shown in Figure 1b. Figure 1c shows the surface morphology of the O_2_-plasma-treated PTFE film, which was generally flat (inset) with uniform micropores. These micropores are conducive to trapping more charges when contacted. Figure 1d shows a schematic plot of rotor and stator in impact and after impact. First, the rotor periodically contacted the stator during rotation. Then, flapping occurred as both rotor and stator deformed and rubbed against each other when the rotor was still forced to rotate. Third, after flapping, the rotor and the stator separated and waited for the next contact in rotation, with the stator in self-vibration. Figure 1e shows a photo of the assembled FR-TENG with testing rigs. Both stators and rotors can adopt horizontal or vertical patterns in the assembled FR-TENG. The FR-TENG adopted the single electrode working mode. The upper rim of the stator fixture and the connector were aligned through vernier caliper, so the two fixed screws on both the stator fixture and the connector were of the same height due to the designed location. By fixing on the screws, the rotor and stator were aligned in height to guarantee direct impact. For comparison, non-patterned stators and rotors were also adopted, simplified as a solid stator or solid rotor in later sections.

**Figure 2:**
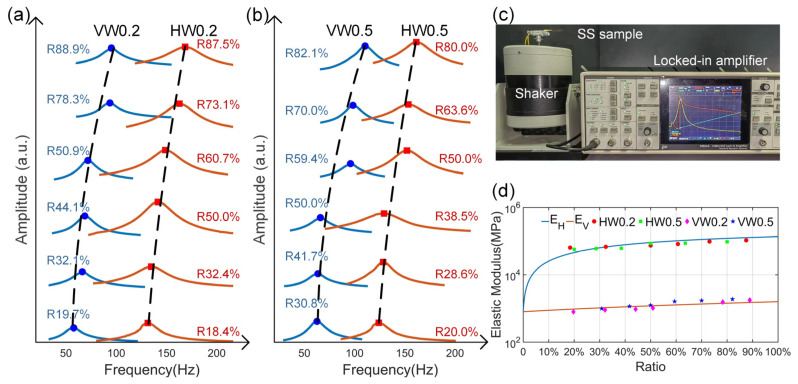
Stiffness modulation effect: (**a**) resonant frequency curves of 12 SS samples with linewidth of 0.2 mm. (**b**) Resonant frequency curves of 12 SS samples with linewidth of 0.5 mm. (**c**) Photo of the resonant frequency testing bench. (**d**) Theoretical (lines) and experimental (dots) elastic modulus comparison for all 24 SS samples.

To prove the effectiveness of the stiffness modulation concept, the resonant frequencies of all 24 SS samples were tested. A layer of polyvinylidene fluoride piezoelectric film was stuck onto the SS samples to transform the tension force into electricity, and the SS samples were mounted on the shaker with changing excitation frequency, as shown in Figure 2c. The resonant frequency curves of the 24 SS samples are shown in Figure 2a,b, which ranged from approximately 57.76 to 178.13 Hz. We denominated each SS sample based on pattern direction (H/V), linewidth (W), and volume ratio (R), so that HW0.2R18.4% represents an SS sample with a horizontal direction, linewidth of 0.2 mm, and volume ratio of 18.4%. The resonant frequency of each SS sample corresponded to the frequency at which the amplitude of the electrical signal reached the maximum. Generally, for each linewidth, resonant frequency increased as the volume ratio increased. For the same linewidth, SS samples with vertical patterns exhibited a lower resonant frequency than those with horizontal patterns. This is in accordance with Equations (2) and (3), as the elastic modulus *E_V_* is lower than *E_H_*. Figure 2d compares the theoretical and experimental elastic moduli for all 24 SS samples, which agree well with each other. Slight deviations might have been due to measurement errors and the additional stiffness effect caused by the stuck piezoelectric layer.

### 3.2. Electrical Output Characteristics

**Figure 3:**
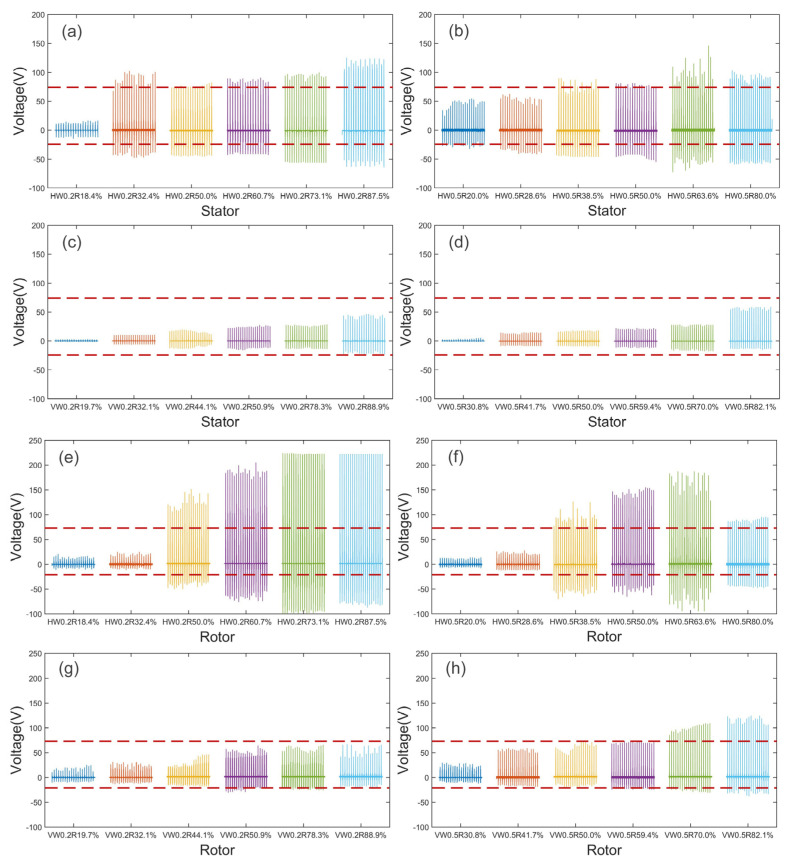
Output voltage curves of stiffness-modulated FR-TENG: (**a**–**d**) solid rotor vs. various SS–PTFE stators; (**e**–**h**) solid stator vs. various SS/PTFE rotors. (**a**,**e**) HW0.2 samples; (**b**,**f**) HW0.5 samples; (**c**,**g**) VW0.2 samples; (**d**,**h**) VW0.5 samples. For comparison, the dashed lines in all subplots show the voltage range generated by solid stator vs. solid rotor.

At a rotation speed of 575 rpm, the voltage generated by different patterned SS/PTFE either as the stator or as the rotor was measured, as shown in Figure 3. For comparison, the output voltage range using non-patterned samples (i.e., solid SS/PTFE stator and solid SS rotor) is also shown, marked by the dashed lines. Figure 3a–d illustrate the comparison of output voltage when employing SS/PTFE samples as stators. The general trend is that output voltage increases as volume ratio increases, and SS/PTFE stators with vertical patterns generate much lower voltage than those with horizontal patterns. Compared with the non-patterned case, the voltage range of all 12 vertical SS/PTFE stators failed to exceed the dashed reference, regardless of linewidth and volume ratio, while 9 of 12 horizontal SS/PTFE stators generated voltage ranges exceeding the dashed reference. This can be explained by the stiffness difference, as vertical patterns result in lower stiffness, lower contact force, and thus reduced triboelectrification effects. Therefore, the horizontal SS/PTFE samples were more suitable for stators. Note that the increasing trend in the peak-to-peak voltage (Vpp) might not continue as *R* approaches infinity, i.e., for the solid SS/PTFE stator, since the voltage range within the dashed lines falls lower than both HW0.2R87.5% and HW0.5R80.0%. This proves that optimizing the volume ratio *R* generates optimal output voltage, demonstrating the effectiveness of stiffness modulation for enhancing power.

Similarly, Figure 3e–h compare the output voltage when employing SS samples as rotors. The voltage generated by the vertical SS samples as rotors was slightly higher than as stators but was still much lower than that of their horizontal counterparts. For the horizontal SS/PTFE rotors with the same linewidth, the *R* is value was critical for output voltage. As shown in Figure 3e,f, the optimal *R* value was 73.1% for the HW0.2 samples and 63.6% for the HW0.5 samples, and the Vpp ranges were 3.32 times and 2.36 times that of the dashed reference, respectively. Note that the voltage limit of the data acquisition system clamped the positive peaks of HW0.2R73.1% and HW0.2R87.5% to 220 V, as shown in Figure 3e, and the optimal *R* value in these two cases was determined by the negative voltage peaks.

**Figure 4:**
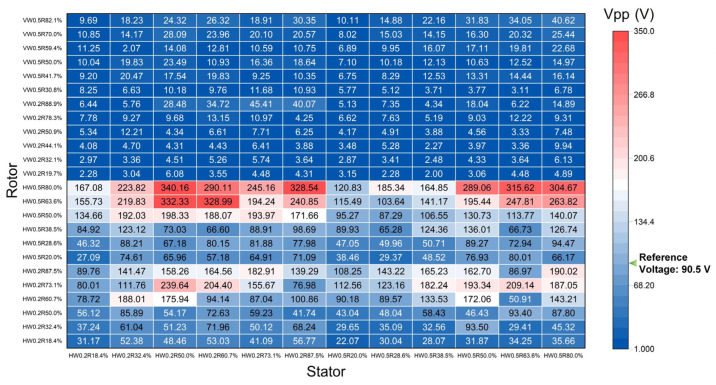
Cross-referenced peak-to-peak voltage generated by all 24 SS/PTFE rotors with 12 horizontal SS/PTFE stators. The reference voltage is 90.5 V.

Based on the above results, horizontal SS/PTFE samples were selected as the stator, and they were rubbed against all SS rotors. Figure 4 summarizes the generated Vpp by cross-referencing the 24 SS rotors with the 12 horizontal SS/PTFE stators. All peak-to-peak voltages were measured at the rotation speed of 575 rpm. Still, the reference Vpp of 90.5 V was obtained for the non-patterned case. Key observations included the following: (1) In the upper half of Figure 4, vertical SS rotors vs. all SS/PTFE stators generate lower output voltage compared with the reference, indicating that the modulated stiffness of the vertical SS/PTFE samples was too low for effective power enhancement; (2) the lower half of Figure 4 shows that the Vpp generated by horizontal SS rotors varied in the wide range of [22.07 V, 340.16 V], corresponding to the 24.39~375.87% referenced to the non-patterned case, which proves the necessity and efficacy of stiffness modulation. (3) Specifically, for HW0.2 rotors, the maximal Vpp mostly occurred at *R* = 73.1~87.5%. For HW0.5 rotors, the maximal Vpp occurred mostly at the largest *R* = 80.0%. For HW0.2 stators, the maximal Vpp occurred for 32.4~50.0%, while for HW0.5 stators, the optimal *R* range was 50.0~80.0%. It can be seen that the rotors require relatively large equivalent stiffness to guarantee a large contact force, while stators require optimized equivalent stiffness. The optimal *R* value is also dependent on the specific rotor–stator combination. (4) The maximum Vpp of 340.16 V was obtained when the stator was HW0.2R50.0%, and the rotor was HW0.5R80.0%, which almost quadruples (3.76 times) the referenced 90.5 V through stiffness modulation. In terms of output power, this corresponds to 14 times improvement compared with the referenced value.

**Figure 5:**
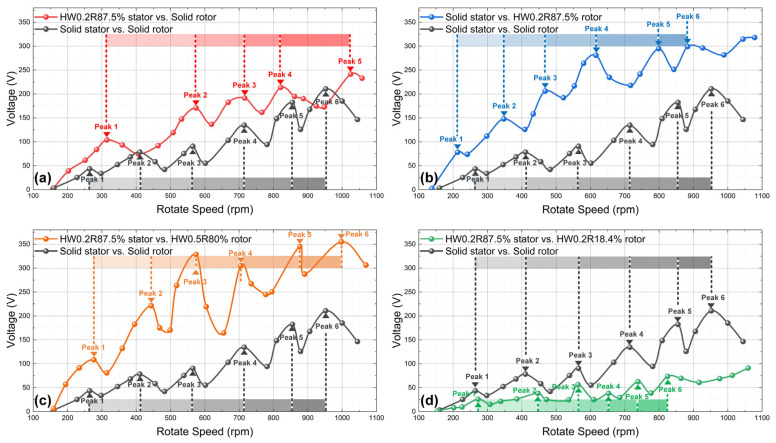
Oscillating voltage-rotation speed relationships for different SSPTFE combinations: (**a**) HW0. 87.5% vs. untreated SS; (**b**) untreated SS vs. HW0.2R87.5%; (**c**) HW0.2R87.5% vs. HW0.5R80%; (**d**) HW0.2R87.5% vs. HW0.2R18.4%. In all four subplots, the dark grey line is generated by solid rotor and solid stator for comparison.

A further reason for the optimal stator *R* variation in Figure 4 was the oscillating relationship between Vpp and rotation speed (Note S1). Since flexible SS/PTFE stators undergo self-oscillation after collision, the original contact state cannot be maintained at the next contact. This results in periodic changes in the actual deflection state and thus periodic changes in output voltage. For the stiffness-modulated FR-TENG, this oscillating relationship was recorded for different combinations of SS/PTFE samples, as shown in Figure 5. For comparison, the oscillating Vpp generated by the solid rotor and solid stator is shown by the dark grey line in all four subplots, representing the control line. For all rotor–stator combinations, Vpp increased with the increase in rotation speed in a fluctuating way. In Figure 5a, the Vpp generated by HW0.2R87.5% stator vs. solid rotor is close to the control line. However, in Figure 5b,c, the Vpp generated by the two combinations of solid stator vs. HW0.2R87.5% rotor and HW0.2R87.5% stator vs. HW0.5R80% rotor is significantly higher than the control line, while, in Figure 5d, the Vpp generated by HW0.2R87.5% stator vs. HW0.2R18.4% rotor is lower than the control line. These results also prove that the stiffness modulation is effective for all rotation speeds, and carefully chosen rotor–stator pairs generate increased power. Another interesting point is that the peak intervals are different for all five curves. This is also because of the stiffness modulation effect, as each stator is of unique resonant frequency and exhibits a unique self-oscillation period, which results in different peaks and peak intervals. The changing rules of the peak intervals for different SS/PTFE samples are worth further investigation.

**Figure 6:**
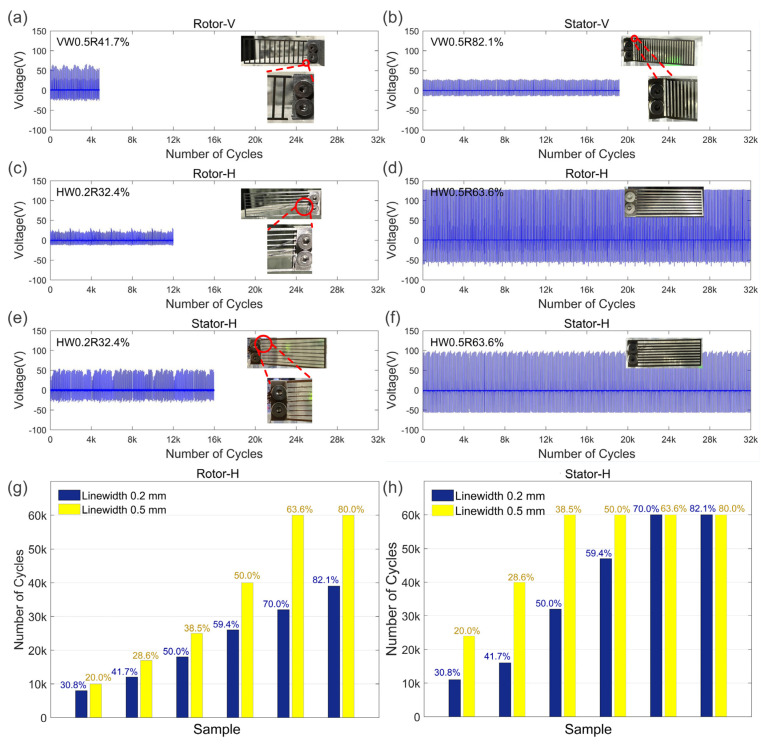
Reliability tests of our different SS/PTFE samples: (**a**) vertical SS rotor; (**b**) vertical SS/PTFE stator; (**c**,**d**) horizontal SS rotor; (**e**,**f**) horizontal SS/PTFE stators; (**g**) number of cycles vs. volume ratio for horizontal SS rotors; (**h**) number of cycles vs. volume ratio for horizontal SS/PTFE stators. The linewidth is 0.2 mm in (**c**,**e**) and 0.5 mm in the remaining photos. The red circles and red lines in (**a**–**c**,**e**) indicate the fracture details.

Reliability tests were performed to specify the durability of different SS/PTFE samples. As shown in Figure 6a,b, because of the force direction, vertical samples were of relatively lower reliability whether utilized as rotor or stator. Figure 6c–f show that the reliability of the horizontal samples was susceptible to both linewidth and volume ratio. For instance, in Figure 6c, the HW0.2R32.4% sample breaks at 12,000 cycles as the rotor, and, in Figure 6e, it breaks at 16,000 cycles as the stator. However, for the HW0.5R63.6% sample, steady output voltage was obtained throughout the tested cycles whether utilized as rotor or stator. Figure 6g,h further quantify the number of cycles for horizontal samples with different linewidths and *R* values. Overall, increasing the volume ratio and linewidth was conducive to enhancing reliability of the FR-TENG. For the same sample, acting as the stator achieved a greater number of cycles than as the rotor, which indicates that rotors endure larger contact force. For a linewidth of 0.2 mm, only stators with *R* ≥ 70.0% survived the tested 60,000 cycles. Meanwhile, for a linewidth of 0.5 mm, both stators with *R* ≥ 33.5% and rotors with *R* ≥ 63.6% survived the tested 60,000 cycles.

### 3.3. Application

**Figure 7:**
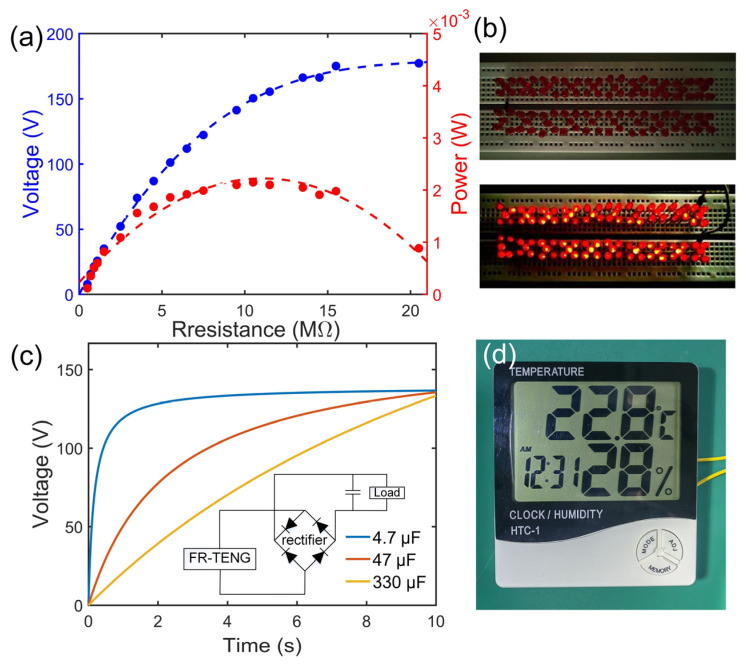
Application of FR-TENG: (**a**) impedance characteristics; (**b**) lightening of 100 LEDs; (**c**) charging curves with different filter capacitors; (**d**) powering a commercial thermo-hygrometer.

Figure 7a shows the impedance characteristics of the FR-TENG. For one pair of rotor and stator, the maximal peak power reached 2.25 mW with an optimal resistance of 10 MΩ. Given the contact area of 1.2 cm^2^, this corresponds to a maximal power density of 18.75 W/m^2^. Table 1 compares the power density of this work with references at different frequencies, where the achieved power density is even higher than most rigid–rigid interfaces. Figure 7b shows that the FR-TENG was used to lighten 100 light-emitting diodes (LEDs) simultaneously (Supplementary Materials Video S1). As shown in Figure 7c, the FR-TENG was utilized in rectifying the application. For the traditional full-bridge circuit, the proposed device could charge 4.7–330 μF filter capacitors to 136 V within 2–10 s. In addition, the electric energy from the FR-TENG could also be used to power a commercial thermo-hygrometer fulfilling all functions in Figure 7d (Supplementary Materials Video S2).

## 4. Conclusions

Inspired by the microstructure of fiber/matrix composite material, an FR-TENG with modulated stiffness was realized with high contact force, high output power, and high reliability. The stiffness of the rotor/stator cantilevers can be adjusted by the direction, linewidth, and volume ratio of micromachined patterns. The resonant frequency of the rotor/stator cantilevers ranges in 57.76~178.13 Hz, corresponding to an effective elastic modulus range of 10^3^~10^5^ MPa. For different rotor–stator pairs, output voltage was modulated from approximately 24.39% to 375.87% compared to the untreated pairs, corresponding to a maximal 14 times increase in output power. For horizontal patterns, high reliability was preserved when volume ratio exceeded 70.0% for 0.2 mm linewidth stators, 33.5% for 0.5 mm linewidth stators, and 63.6% for 0.5 mm linewidth rotors. Oscillating relationships between output voltage and rotation speed were observed for different rotor–stator pairs. The FR-TENG with one rotor–stator pair achieved a maximal power density of 18.75 W/m^2^ and was able to lighten 100 LEDs and power a commercial thermo-hygrometer performing all functions. The stiffness modulation method provides a new way to improve both the power and reliability of flexible rotational triboelectric nanogenerators.

## Figures and Tables

**Table 1:** Comparison of peak power density between references and this work.

References	Rigid/Flexible Interfaces	Frequency (Hz)	Peak Power Density (W/m^2^)
[1]	Rigid–rigid	5.6	7.74 ^a^
[5]	Rigid–rigid	6.7	1.91 ^a^
[7]	Rigid–rigid	2	3.25
[11]	Rigid–rigid	10	2.28
[26]	Rigid–flexible	16.7	0.002 ^a^
[27]	Rigid–flexible	13	0.0004 ^a^
[28]	Rigid–flexible	4	0.58 ^a^
[31]	Rigid–flexible	5.4	4.4
[17]	Flexible–flexible	2.5	0.02 ^a^
[29]	Flexible–flexible	6	0.64 ^a^
[36]	Flexible–flexible	1	0.3
[37]	Flexible–flexible	3	0.8
**This Work**	**Flexible–flexible**	**9.6**	**18.75**

^a^ Estimated from data in the reference.

## Data Availability

Dataset available on request from the authors.

## Supplementary Materials

The following supporting information can be downloaded at: https://www.mdpi.com/article/10.3390/nano14040380/s1, Note S1. The explanation of the oscillating relationship between Vpp and rotation speed (Note S1) (PDF); Video S1. lightening of 100 LEDs (MP4); Video S2. powering a commercial thermo-hygrometer (MP4).
