# Supplementary material for: Stiffness Modulation in Flexible Rotational Triboelectric Nanogenerators for Dual Enhancement of Power and Reliability
Source: Nanomaterials (Basel). 2024 Feb 18;14(4):380. doi: 10.3390/nano14040380 (PMC10892494; doi:10.3390/nano14040380)
Supplement: Supplementary file 1 [file nanomaterials-14-00380-s001.zip › Note S1- Explanation of oscillating relation.pdf]

FR-TENG is designed based on the rotor stator pairs (as shown in Fig.S1.a), where f_S and f respectively represent the stator natural frequency and rotor rotation frequency. These two parameters can be represented by the self-oscillation period (T_S) and rotation period (T), respectively $T_S = 1/f_S$ and $T = 1/f$. L is the overlap length and R is the working radius of the device. Fig.S1.b shows the dynamic process of the flexible SS/PTFE sample during operation. The flexible stator is set to be in equilibrium state at the beginning (zero deflection and zero velocity), and the rotor is continuously approaching the flexible stator from (i) pre-contact state. After contact, in (ii) sliding state, charge pairs are generated on the interface between the dielectric layer on the stator electrode and the rotor electrode, and voltage spikes are generated on the external load. This is followed by the (iii) separation and oscillation state, at which point the rotor passes and the flexible stator begins to self-oscillate at its natural frequency. In addition, the "contact slip separation" process of the flexible SS/PTFE specimen during the working process is verified in the Fig.S1.c, which shows the motion states of the sample captured by the high-speed camera. The rotor is separated from the stator in the initial state i, the rotor is in contact with the stator and charge transfer occurs as the rotor reaches state ii, the rotor and the stator slip in a flexible way as the rotor continues to rotate, as shown in State iii, and finally the rotor and the stator are separated at the tip (state iv).

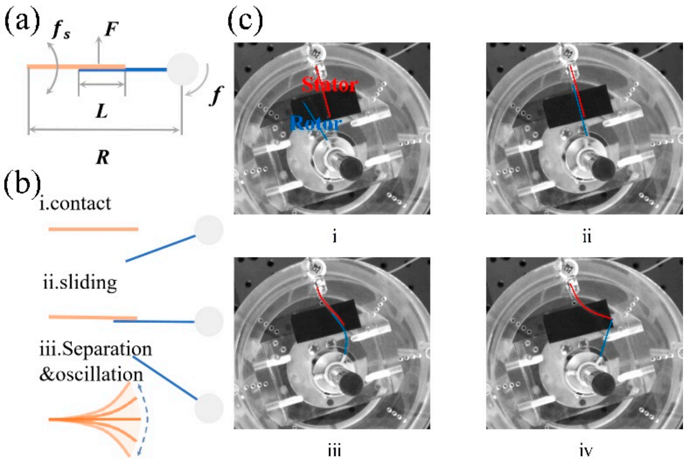

Figure S1. (a) schematic diagram of R-TENG; (b) contact-slide-separation mechanism; (c) Dynamic characteristics captured by high-speed cameras.

It is this self-oscillating process that causes the output fluctuations of the FR-TENG. As shown in Fig.S2.a, When the flexible stator is subjected to external force F, the stator deformation occurs, and the stator is subjected to inertial force $m\ddot{x}$, damping force $\xi\dot{x}$ and elastic force kx. According to the principle of force balance, the dynamic equation can be obtained:

$$m\ddot{x}+\xi\dot{x}+kx=F \tag{1-1}$$

where m represents the equivalent mass of flexible stator, ξ represents flexible stator damping ratio, and k represents equivalent spring stiffness of the flexible stator.

Fig.S2.b shows the force acting on flexible stator. In order to facilitate calculation, the contact time t_c between the rotor and the stator is set to a fixed value, and the exciting force $F(t)$ acting on the flexible stator can be obtained as follows:

$$F(t)=\begin{cases}F_A, & 0+nT\le t\le nT+t_c\\ 0, & nT+t_c\le t\le (n+1)T\end{cases} \tag{1-2}$$

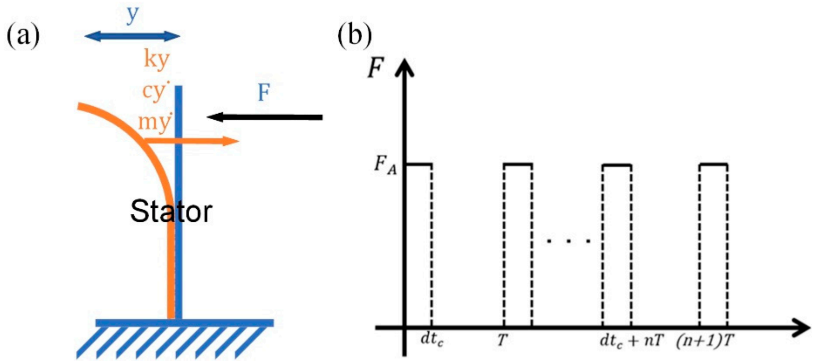

Figure S2. (a) Force analysis of stator blades; (b) Equal force acting on stator blades.

The actual deflection state of the flexible stator can be described by tip displacement and velocity. Before the first collision, the flexible stator is in equilibrium, with an initial displacement $x=0$ and an initial velocity $v=0$. As the rotor rotates and contacts the stator, it generates displacement and velocity. Fig.S3.a shows the stator displacement after a collision in an undamped state. After the collision, if external excitation is no longer applied, the tip displacement of the flexible stator will gradually decrease to 0. However, the actual motion of R-TENG is continuous, which leads to the flexible stator not returning to their initial equilibrium state. Then the next collision occurs, as shown in Fig.S3.b. In the initial state, the stator is tightly attached to the rotor,

but the state where the rotor contacts with the stator again after a collision is not fixed. Therefore, during continuous operation of R-TENG, the location of each stator rotor collision is random.

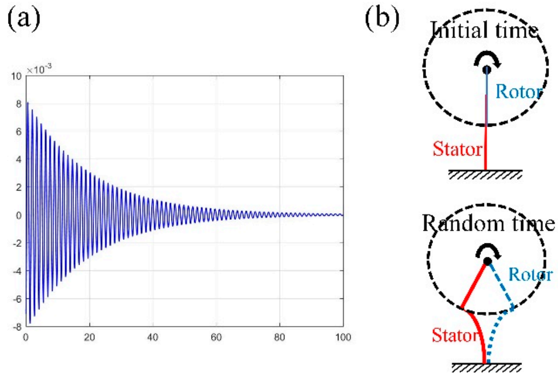

Figure S3. (a) Displacement of stator tip after collision; (b) Disturbance of rotor and stator during collision (initial and random time).

Therefore, the main reason for this phenomenon is the large deformation capacity of the flexible stator, as the spring mechanism causes the flexible stator to oscillate after a single contact. During the self-oscillation process, the deflection and velocity of the flexible stator change, and cannot be guaranteed to return to the original equilibrium state at the next contact, resulting in a change in the actual deflection state and ultimately resulting in an oscillation relation in the output voltage.
